# Devising Assisted Reproductive Technologies for Wild-Derived Strains of Mice: 37 Strains from Five Subspecies of *Mus musculus*

**DOI:** 10.1371/journal.pone.0114305

**Published:** 2014-12-03

**Authors:** Keiji Mochida, Ayumi Hasegawa, Naoki Otaka, Daiki Hama, Takashi Furuya, Masaki Yamaguchi, Eri Ichikawa, Maiko Ijuin, Kyuichi Taguma, Michiko Hashimoto, Rika Takashima, Masayo Kadota, Noriko Hiraiwa, Kazuyuki Mekada, Atsushi Yoshiki, Atsuo Ogura

**Affiliations:** 1 RIKEN BioResouce Center, Tsukuba, Ibaraki, Japan; 2 Graduate School of Life and Environmental Science, University of Tsukuba, Tsukuba, Ibaraki, Japan; 3 Center for Disease Biology and Integrative Medicine, Faculty of Medicine, University of Tokyo, Bunkyo-ku, Tokyo, Japan; Institute of Zoology, Chinese Academy of Sciences, China

## Abstract

Wild-derived mice have long offered invaluable experimental models for mouse genetics because of their high evolutionary divergence from laboratory mice. A number of wild-derived strains are available from the RIKEN BioResource Center (BRC), but they have been maintained as living stocks because of the unavailability of assisted reproductive technology (ART). In this study, we sought to devise ART for 37 wild-derived strains from five subspecies of *Mus musculus* maintained at the BRC. Superovulation of females was effective (more than 15 oocytes per female) for 34 out of 37 strains by treatment with either equine chorionic gonadotropin or anti-inhibin serum, depending on their genetic background (subspecies). The collected oocytes could be fertilized *in vitro* at mean rates of 79.0% and 54.6% by the optimized protocol using fresh or frozen-thawed spermatozoa, respectively. They were cryopreserved at the 2-cell stage by vitrification with an ethylene glycol-based solution. In total, 94.6% of cryopreserved embryos survived the vitrification procedure and restored their normal morphology after warming. A conventional embryo transfer protocol could be applied to 25 out of the 35 strains tested. In the remaining 10 strains, live offspring could be obtained by a modified embryo transfer protocol using cyclosporin A treatment and co-transfer of ICR (laboratory mouse strain) embryos. Thus, ART for 37 wild-derived strains was devised successfully and is now routinely used for their preservation and transportation. The information provided here might facilitate broader use and wider distribution of wild-derived mice for biomedical research.

## Introduction

Laboratory mice (*Mus musculus*) are the most frequently used mammals for biomedical research because of their defined genetic background and the ease of doing genetic modifications [1]. Undoubtedly, their contributions to the development of basic life science and invention of new biotechnologies are enormous. However, the genetic diversity of the classical laboratory mice is thought to be limited because they were derived from a relatively small pool of “fancy” mice with genetic backgrounds composed predominantly of the Western European subspecies *M. musculus domesticus* and the Japanese subspecies *M. musculus molossinus*, specifically JF1/Ms [2]. Therefore, many efforts have been made to increase the genetic diversity of mice for genomic research in this species by introducing wild-captured mice into laboratories [3]–[6]. Such newly introduced genetic resources are expected to increase the chance of finding polymorphisms or novel modifier genes that are responsible for some disease-resistant or disease-prone characteristics, providing invaluable information on the genetic bases of corresponding diseases in humans [7]–[9]. Furthermore, a large pool of polymorphisms that discriminate the conventional laboratory mice from wild-derived mice might enable more accurate and more efficient quantitative trait locus (QTL) analyses [10], [11].

The RIKEN BioResource Center (BRC) is one of the major international repository centers of biological research resources (http://en.brc.riken.jp/index.shtml) and has collected over 7,000 mouse strains to date [12]. Besides its large collection of historical inbred strains and newly established gene-modified strains, a large set of wild-derived strains from four species of *Mus* makes the BRC a unique and invaluable core center of mouse genetic resources. These wild-derived strains have been propagated by inbreeding and are maintained under specific-pathogen-free conditions. However, such strain maintenance as living stocks under strict genetic and microbiological conditions costs much money and effort; mice of most wild-derived strains move very quickly and can jump high. Distribution of such live mice to other facilities also requires intense care to avoid accidental escapes during transportation. To overcome these problems associated with maintenance and distribution of wild-derived strains, we have recently developed a series of basic assisted reproductive technologies (ARTs) for two Japanese wild-derived strains, MSM/Ms and JF1/Ms. Indeed, the efficient production of cryopreserved embryos of these wild-derived strains has enabled safer and cheaper strain preservation and transportation without the risk of escapes. The major technical advancements we made were efficient superovulation by treatment with anti-inhibin serum in the MSM and JF1 strains, and avoidance of intrauterine deaths of fetuses in recipient ICR females for the MSM strain [13]. The combination of optimally devised ARTs has enabled us to store embryos from JF1 and MSM in liquid nitrogen safely and to restore live mice from these cryopreserved embryos as necessary. Because our ultimate goal is generating a complete set of cryostocks from all the wild strains maintained at the BRC, our next step is to optimize every ART procedure for each of these different strains. In this study, we sought to find the best protocols for superovulation, *in vitro* fertilization (IVF), embryo cryopreservation, and embryo transfer using 37 strains from five subspecies of *Mus musculus* maintained at the BRC. As a result, we successfully devised ART protocols for each strain, attaining practical efficiencies at each step. The large set of our ART data using 37 wild-derived strains will enable the broader use of these invaluable genetic resources and facilitate further developments in the field of mouse genetics.

## Materials and Methods

### Animals

Thirty-seven strains of wild-derived mice used in this study were provided by the RIKEN BRC (Table 1). The MSM-Tyr^c^ and MSM-W^v^ strains are MSM-background congenic strains carrying mutant alleles found in laboratory strains. Therefore, they are not wild-derived strains in a strict sense, but are classified into *M. m. molossinus* in this study conveniently. Standard inbred strains of mice, C57BL/6JJcl, C57BL/6NJcl, BALB/cAJcl, C3H/HeJJcl (CLEA Japan Inc., Tokyo, Japan), 129X1/SvJJmsSlc (SLC Co. Ltd., Shizuoka, Japan), DBA/2NCrlCrlj (Charles River Laboratories Japan, Inc., Tokyo, Japan) were also used for comparisons. For embryo transfer experiments, female ICR mice (CLEA Japan Inc.) were used as pseudopregnant recipients after being mated with vasectomized male ICR mice. All mice were maintained under specific-pathogen-free conditions, provided with water and commercial laboratory mouse chow ad libitum, and housed under controlled lighting conditions (daily light period, 08∶00–20∶00). On the day of experiments, animals were sacrificed by cervical dislocation and used for experiments described below. All animal experiments described here were approved by the Animal Experimentation Committee at the RIKEN Tsukuba Institute and were performed in accordance with the committee’s guiding principles.

**Table 1:** List of the strain name and the origin in wild-derived strains.

Strain		Abbreviation	Origin	Strain number in BRC	Name of Depositor
***M. m. molossinus***					
	MSM/MsRbrc	MSM	Mishima, Japan	RBRC00209	Toshihiko Shiroishi
	JF1/MsRbrc	JF1	Japan	RBRC00639	Toshihiko Shiroishi
	MOM/NgaRbrc	MOM	Nagoya, Japan	RBRC01837	Takao Namikawa
	AIZ/StmRbrc	AIZ	Aizu, Japan	RBRC00430	Yoshibumi Matsushima
	MAE/StmRbrc	MAE	Maezawa, Japan	RBRC00431	Yoshibumi Matsushima
	KOR1/StmRbrc	KOR1	Koriyama, Japan	RBRC00427	Yoshibumi Matsushima
	KOR5/StmRbrc	KOR5	Koriyama, Japan	RBRC00428	Yoshibumi Matsushima
	KOR7/StmRbrc	KOR7	Koriyama, Japan	RBRC00429	Yoshibumi Matsushima
	STM1/StmRbrc	STM1	Saitama, Japan	RBRC00265	Yoshibumi Matsushima
	STM2/StmRbrc	STM2	Saitama, Japan	RBRC00266	Yoshibumi Matsushima
	KOR1-Is^Stm^/StmRbrc	KOR1-Is	(KOR1 carrying a spontaneous mutation)	RBRC00436	Yoshibumi Matsushima
	*MSM/Ms-Tyr^c^/RinRbrc	MSM-Tyr^c^	(MSM background)	RBRC01770	Hiromichi Yonekawa
	*MSM.NC-KitW^−v^/OhnotRbrc	MSM-W^v^	(MSM background)	RBRC01140	Tamio Ohno
***M. m. musculus***					
	PWK/RpRbrc	PWK	Czechoslovakia	RBRC00213	Roswell Park Cancer Institute
	Kaz/MzTuaRbrc	KAZ	Kazakhstan	RBRC01237	Kimiyuki Tsuchiya
	GOR/MzTuaRbrc	GOR	Gorno-Altaysk, Russia	RBRC01242	Kimiyuki Tsuchiya
	Tom/MzTuaRbrc	TOM	Tomsk, Tuva, West Siberia, Russia	RBRC01243	Kimiyuki Tsuchiya
	OKH/MzTuaRbrc	OKH	Okha, Russia	RBRC01245	Kimiyuki Tsuchiya
	Knb/MzTuaRbrc	KNB	Balkhash Lake, Kazakhstan	RBRC01247	Kimiyuki Tsuchiya
	AST/MzTuaRbrc	AST	Astrakhan, Russia	RBRC01249	Kimiyuki Tsuchiya
	AST(t−)/MzTuaRbrc	AST(t−)	(AST carrying a spontaneous mutation)	RBRC01341	Kimiyuki Tsuchiya
	AKT/MzTuaRbrc	AKT	Aktybinsk, Kazakhstan	RBRC01238	Kimiyuki Tsuchiya
	Irk/MzTuaRbrc	IRK	Irkutsk, East Siberia, Russia	RBRC01241	Kimiyuki Tsuchiya
	NJL/MsRbrc	NJL	Northern Jetland, Denmark	RBRC00207	Toshihiko Shiroishi
	MBT/PasRbrc	MBT	Bulgaria	RBRC01164	Jean-Louis Guenet
	BLG2/MsRbrc	BLG2	Toshevo, Bulgaria	RBRC00653	Toshihiko Shiroishi
***M. m. domesticus***					
	WLA/PasRbrc	WLA	Toulouse, France	RBRC01168	Xavier Montagutelli
	WMP/PasRbrc	WMP	Monastir, Tunigia	RBRC01167	Xavier Montagutelli
	BFM/2MsRbrc	BFM/2	Montpellier, France	RBRC00659	Toshihiko Shiroishi
	PGN2/MsRbrc	PGN2	Pegion, Canada	RBRC00667	Toshihiko Shiroishi
***M. m. castaneus***					
	HMI/MsRbrc	HMI	Hemei, Taiwan	RBRC00657	Toshihiko Shiroishi
	MYS/MzRbrc	MYS	Mysore, India	RBRC01196	Chihiro Koshimoto [30]
	CASP/1NgaRbrc	CASP	Los Banos, Philippines	RBRC03108	Yoichi Matsuda
***M. m. spp.***					
*(M. m. yamasinai)*	SWN/MsRbrc	SWN	Suwon, South Korea	RBRC00654	Toshihiko Shiroishi
	KJR/MsRbrc	KJR	Kojuri, South Korea	RBRC00655	Toshihiko Shiroishi
*(M. m. gansuensis)*	CHD/MsRbrc	CHD	Chengdu, China	RBRC00738	Toshihiko Shiroishi
*(unknown)*	JIL/OdaRbrc	JIL	Jilin, China	RBRC00841	Senichi Oda

*MSM-Tyr^c^ and MSM-W^v^ strains are congenic strains carrying Tyr^c^ (albino) and KitW^−v^ mutations originating from laboratory strains, respectively. They have been repeatedly backcrossed to MSM eight and 15 times, respectively.

### Superovulation

Female mice from wild-derived strains at 4 to 10 weeks of age were injected intraperitoneally with 5 IU equine chorionic gonadotropin (eCG; Peamex, Sankyo Co., Tokyo, Japan) or anti-inhibin serum (AIS, 100 µl per female), followed by an injection of 7.5 IU human chorionic gonadotropin (hCG; Puberogen, Sankyo) 48 h later. Sixteen to 18 h after hCG injection, mature metaphase II (MII) oocytes were collected from oviducts and ovarian follicles. Oviductal (ovulated) oocytes were collected from oviducts by puncturing the ampulla region with a 26–27 G needle under mineral oil. Ovarian oocytes with expanded cumulus cells were collected into PB1 medium [14] under mineral oil by puncturing intact full-size follicles on the ovarian surface. Oocytes collected from the oviducts or ovarian follicles were moved to fresh medium. Abnormal oviductal oocytes were discernible either at 3–5 h or at 22–25 h after insemination by their deformed contours or cytoplasmic fragmentation. The AIS was obtained from a castrated goat immunized against [Tyr30]-inhibin α (l-30) NH_2_ conjugated to rabbit serum albumin as reported [15].

### IVF

IVF was performed as previously described with slight modifications [13]. In brief, human tubal fluid (HTF) medium [16] containing hypotaurine (0.11 mg/ml, Sigma-Aldrich, St Louis, MO, USA) and 0.3% bovine serum albumin (BSA, Calbiochem, Merck Millipore, Darmstadt, Germany) was used as the basic medium for sperm preincubation and fertilization. Spermatozoa from the epididymal caudae of male mice (3–15 months old) from each strain were suspended in 200 µl of sperm preincubation medium (HTF containing 0.4 mM methyl-β-cyclodextrin [17], [18] and 0.1 mg/ml polyvinyl alcohol instead of BSA) and incubated at 37°C under 5% CO_2_ in air for 45–60 min. Spermatozoa that had been frozen in plastic straws were thawed and preincubated as described previously until used for IVF [19]. At the time of insemination, the preincubated spermatozoa were transferred into 80 µl drops of fertilization medium containing cumulus-enclosed oocytes at a final concentration of 200–500 spermatozoa/µl. The fertilization medium was basically the HTF medium described above, but when frozen-thawed spermatozoa were used or the fertilization rates were less than 50% with fresh samples, 1.25 mM reduced glutathione (GSH) [20], [21] was added. At 4 to 5 h after insemination, spermatozoa and cumulus cells were removed from the oocytes by gentle pipetting. After washing with fresh medium, oocytes were cultured in CZB medium [22] containing 5.6 mM glucose, 0.1 mg/ml polyvinyl alcohol and 3.0 mg/ml BSA, and cultured at 37°C under 5% CO_2_ in air for approximately 24 h. Oocytes that developed into normal-appearing 2-cell embryos were considered fertilized.

### Embryo Cryopreservation by Vitrification

We cryopreserved 2-cell embryos–mainly produced by IVF–using high osmolality vitrification (HOV) solution (ARK Resource Inc., Kumamoto, Japan). This solution was originally developed in our laboratory to increase the survivability of vitrified embryos at the temperature of dry ice (around –80°C) [23]. In brief, about 20–30 embryos were placed on the surface of a 50 µl droplet of equilibrium solution (5% dimethyl sulfoxide and 5% ethylene glycol in PB1) in air using a sterile glass capillary (100–120 µm diameter) with a small amount of medium at room temperature. After 3 min, embryos that had settled on the bottom were picked up and transferred into a cryotube (MS-4501; Sumitomo Bakelite Co. Ltd., Tokyo, Japan) containing 50 µl of the vitrification solution: 42.5% (v/v) ethylene glycol, 17.3% (w/v) Ficoll and 1.0 M sucrose in PB1. After 1 min, the cryotube was plunged directly into LN_2_ at –196°C. On the day of embryo transfer, the cryotubes were retrieved from LN_2_ and the cap was quickly removed to allow LN_2_ to evaporate from the tubes. After they had been left at room temperature for 2 min, 850 µl of 0.75 M sucrose-PB1 was gently added to the tubes. After 4 min, the solution in the tubes was mixed by gentle pipetting five times. Then the entire volume of the solution was transferred to an empty plastic dish. After 1–2 min, the embryos were picked up and transferred into a drop of 0.25 M sucrose in PB1. After equilibration for another 1–2 min, the recovered embryos were washed in another two drops of 0.25 M sucrose in PB1 and then transferred to a drop of CZB medium in a culture dish. Embryos with normal morphology (i.e., with an intact plasma membrane and clear cytoplasm) were considered viable. The surviving embryos were incubated under 5% CO_2_ in air at 37°C until embryo transfer (<3 h).

### Embryo Transfer

Vitrified–recovered embryos were transferred into the oviducts of day 1 pseudopregnant females by either of the following two methods [13]. The first was a conventional one, in which about six embryos (range, four to nine) from wild-derived mice were transferred into each oviduct of an ICR recipient. The other was an improved protocol, in which about three embryos (two to six) of wild-derived mice mixed with three IVF-derived ICR embryos were transferred into each of the oviducts of an ICR recipient. In the latter co-transfer method, recipient females were injected subcutaneously with 1 mg/kg cyclosporine A (CsA) (Sigma-Aldrich) in the morning (10∶00–11∶00) of day 5. All embryo transfer experiments were carried out under appropriate anesthesia. In all experiments, recipient females were each injected subcutaneously with 2 mg progesterone (Progehormone, Mochida Pharmaceutical Co. Ltd, Tokyo, Japan) in the evening on days 18 and 19 to avoid natural delivery. On day 20 (09∶00–12∶00), the recipient females were examined for the presence of fetuses by Caesarian section and live pups were nursed by lactating ICR foster mothers.

### Statistical Analysis

The numbers of oocytes collected from all wild-derived strains of mice were analyzed by two-way analysis of variance (ANOVA) using IBM SPSS Statistics (IBM Corp., Armonk, NY, USA), as appropriate. The Steel–Dwass test was used for multiple comparisons. Other statistical methods were applied appropriately, as indicated in the Results and *P*<0.05 was considered statistically significant.

## Results

### Collection of Oocytes

**Figure 1:**
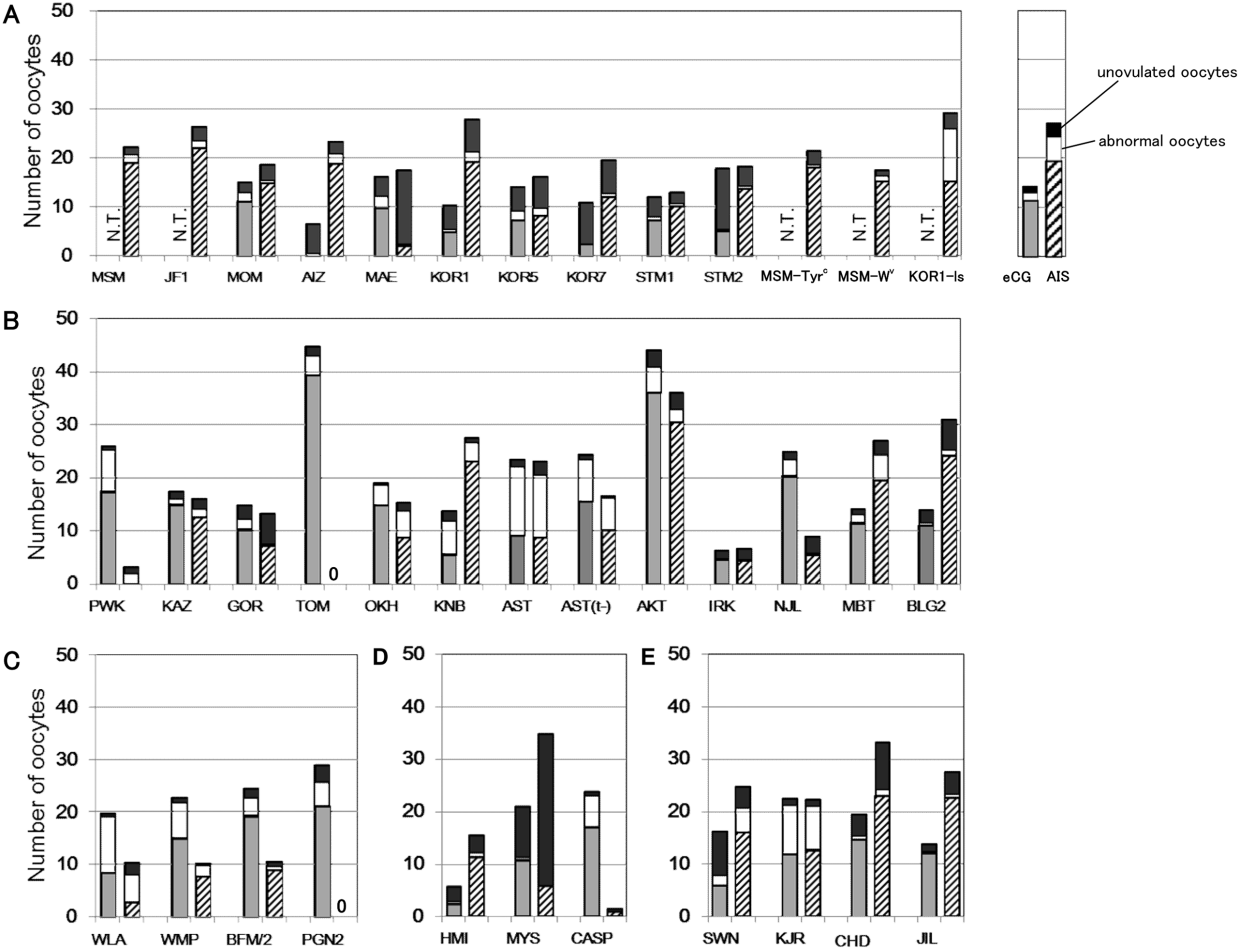
The mean numbers of oocytes collected per female after eCG or AIS treatment. Each bar shows the total number of oocytes consisting of normal ovulated oocytes (gray or hatched), abnormally ovulated oocytes (white), and unovulated ovarian oocytes (black). In 34 out of 37 strains, more than 15 oocytes per female were obtained either by eCG or AIS superovulation treatment. In *M. m. molossinus*, the strains known to be highly poor responders to eCG were not tested for eCG to save the number of animals used (shown as N.T.). The detailed data are summarized in Table S1.

We collected oocytes from 37 wild-derived strains following eCG or AIS treatment (Figure 1, Table S1). In all strains tested, MII oocytes were also obtained from unruptured follicles in the ovaries although their numbers greatly varied according to the strain. In our preliminary experiments, we compared the fertilizability and developmental ability of ovarian oocytes with those of oviductal (ovulated) oocytes and found that the results were indistinguishable (data not shown). Therefore, we did not discriminate ovarian from oviductal oocytes and pooled them for subsequent experiments. The data were obtained from 11–86 females for each strain. In some experiments, because ovarian oocytes were collected from pooled ovaries, the exact number of oocytes collected per female was not recorded. Therefore, the mean number of ovulated oocytes was calculated for each strain and used for the subsequent statistical analysis.

**Figure 2:**
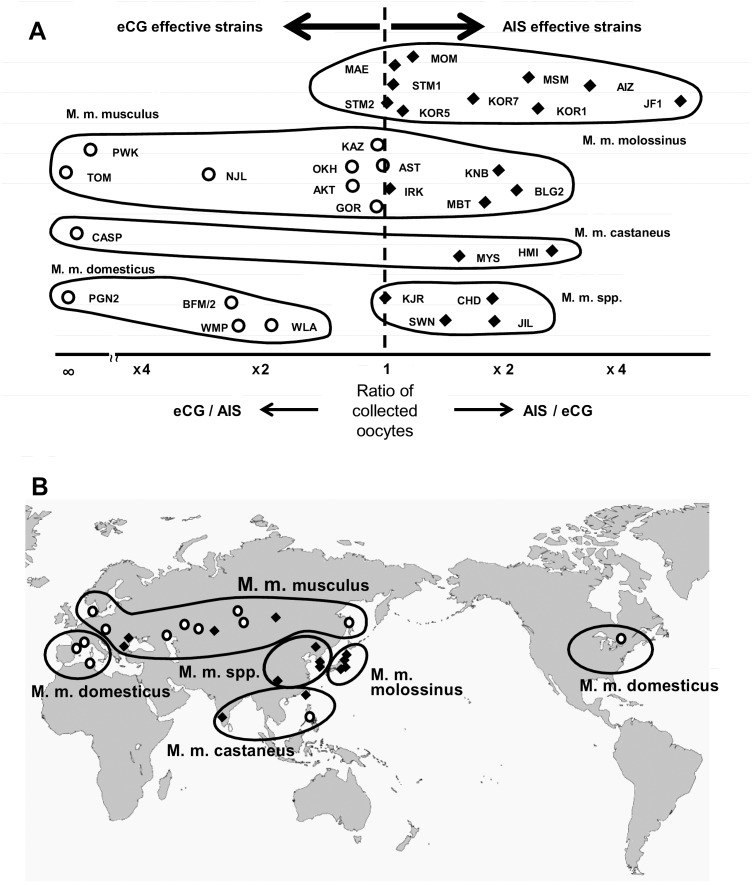
Comparison of the effectiveness of eCG and AIS treatments for superovulation. Strains that responded better to eCG than to AIS are indicated with white circles, and those that responded better to AIS than eCG are indicated with black diamonds. For MSM and JF1, the ratio was calculated from the date reported previously [13]. Other strains without data for eCG (Figure 1) are omitted from this figure. (A) Scatterplots displaying the comparative effectiveness of superovulation with eCG and AIS. The strains are grouped by ovals representing the subspecies so that the subspecies-specific tendencies can be observed. There was a significant effect of the superovulation regimen on the numbers of oocytes collected in *M. m. molossinus*, *M. m. domesticus*, and *M. m. spp* (Table 2). (B) A world map showing the place of origin of wild-derived strains. There was a trend that strains from Asia were more AIS-sensitive and those from Europe and the North America were more eCG-sensitive.

At least 15 oocytes per female on average were collected following eCG or AIS treatment in 34 out of 37 strains, indicating that most strains can be superovulated by selecting the appropriate regimen (Figure 1, Table S1). We then performed statistical analysis to determine which superovulation regimen was most effective for each wild-derived strain. We employed two-way ANOVA consisting of two factors (subspecies and superovulation regimen) using the mean numbers of oocytes ovulated per female in every strain. The mean number of oocytes was significantly affected by the subspecies (*P* = 0.049), but not by the superovulation regimen (*P* = 0.407) (Table 2). There was an interaction between these two factors, indicating that the expected number of oocytes collected was determined by the combination of these factors. However, there were no significant differences between these combinations (5 subspecies×2 superovulation regimens = 10 combinations) according to the multiple comparison Steel–Dwass test. When we performed intra-subspecies comparisons between the eCG and AIS groups (one-way ANOVA), there was a significant effect of the superovulation regimen in three subspecies, *M. m. molossinus*, *M. m. domesticus*, and *M. m. spp* (Table 2). Indeed, when the ratios of the mean numbers of oocytes collected following either eCG or AIS treatments were plotted, we could see a clear bias for eCG or AIS in these three subspecies; AIS was better for *M. m. molossinus* and *M. m. spp*, and eCG was better for *M. m. domesticus* (Figure 2A). By contrast, the results for the remaining two subspecies, *M. m. musculus* and *M. m. castaneus*, were more diverse and there seemed to be strain-specific differences within the subspecies. All the raw data including the ages of females (weeks after birth) can be found in Tables S2, S3, and S4.

**Table 2:** Probabilities (P values) of main effects on the number of ovulated oocytes and their interactions.

Subspecies	Effect and interaction		P value
All*	Main effect	Subspecies	0.049
		Superovulation	0.407
	Interaction between two factors		0.002
M. m. molossinus**	Main effect	Superovulation	0.002
M. m. musculus**	Main effect	Superovulation	0.203
M. m. domesticus**	Main effect	Superovulation	0.001
M. m. castaneus**	Main effect	Superovulation	0.417
M. m. spp.**	Main effect	Superovulation	0.033

Results were obtained by two-way* and one-way** ANOVA analysis.

P<0.05 was considered significant (underlined).

We plotted the natural habitats (i.e., the places of capture) on a world map for the wild-derived strains. There was a tendency that strains from Asia were more AIS-responsive and those from Europe and the North America were more eCG-responsive, although more data are necessary for a conclusive result (Figure 2B).

### IVF with Fresh and Frozen Spermatozoa

**Figure 3:**
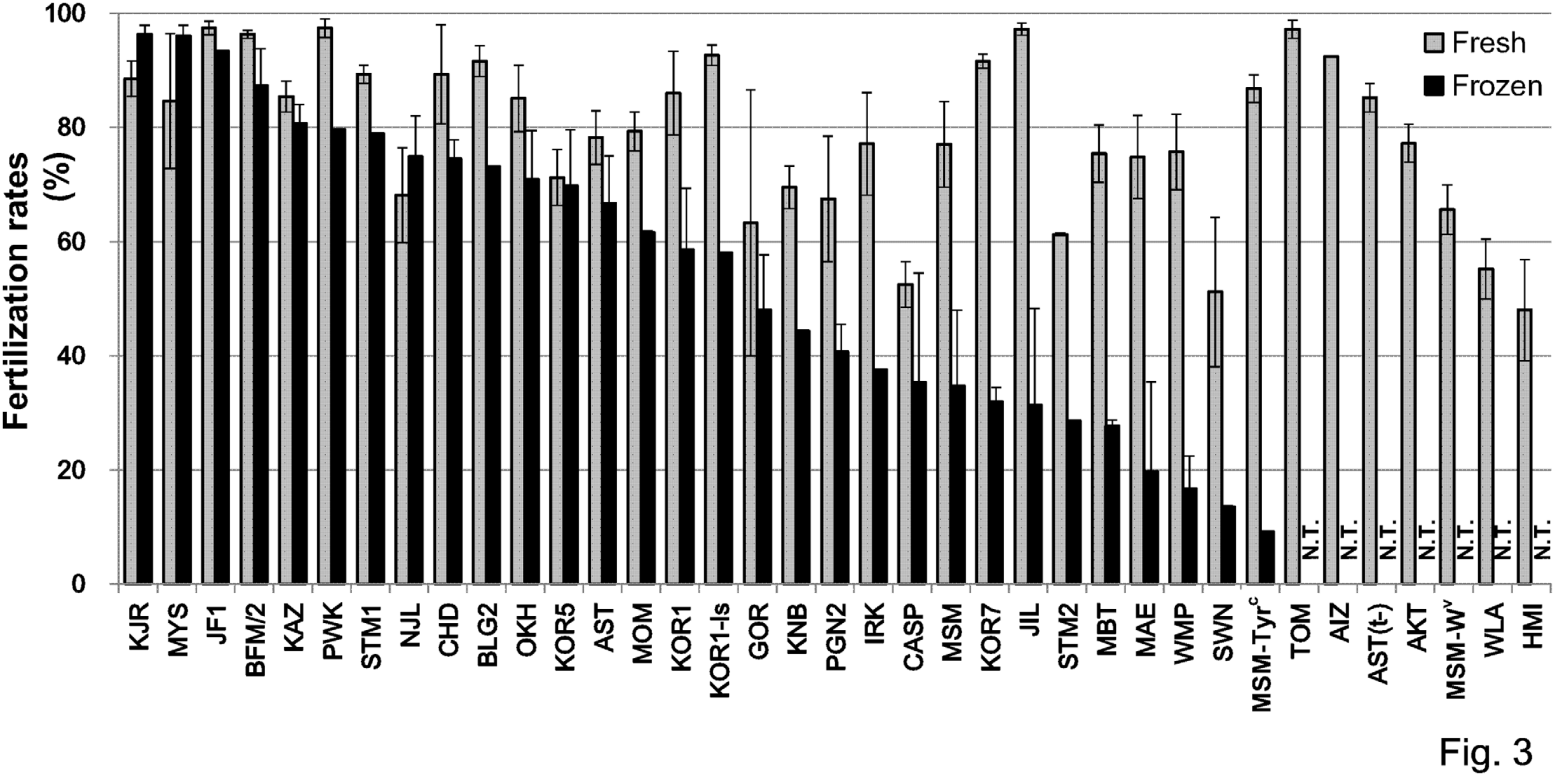
Fertilization rates after IVF with fresh (gray) or frozen (black) spermatozoa. Strains are placed in the order of the fertilization rates using frozen spermatozoa. The detailed data set is available in Table S5. N.T., not tested. Error bars = S.E.M.

We produced fertilized oocytes by IVF with fresh and frozen-thawed spermatozoa from 37 and 30 strains, and the respective mean fertilization rates were 79.0% and 54.6% (Figure 3, Table S5). Fertilization rates of more than 50% were obtained in 33/37 strains (89%) of fresh spermatozoa without the need to use GSH (Table S5). When we compared the fertilization rates with or without GSH in four strains (WLA, WMP, HMI, and CASP), all showed improved fertilization rates, especially for HMI with an increase from 10.9% to 48.0% (Table S5). We added GSH to the fertilization medium when frozen-thawed spermatozoa were used for IVF. Fertilized oocytes could be obtained successfully using frozen spermatozoa in all 30 strains tested, although four of them showed poor fertilization rates of less than 20% (Figure 3, Table S5).

### Cryopreservation and Transfer of Embryos

**Figure 4:**
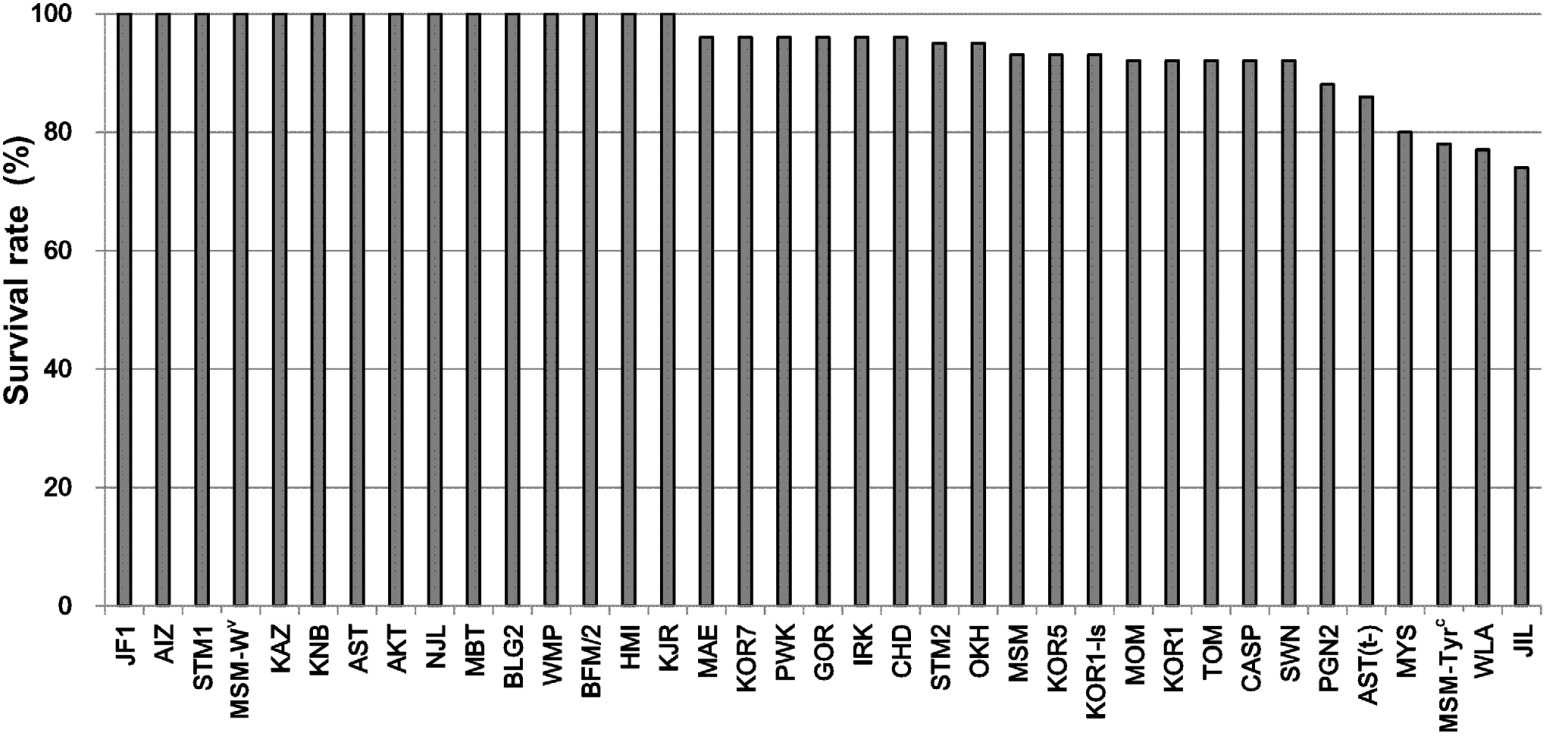
Survival rates of 2-cell embryos after vitrification by HOV and warming. The detailed data set is available in Table S6.

**Figure 5:**
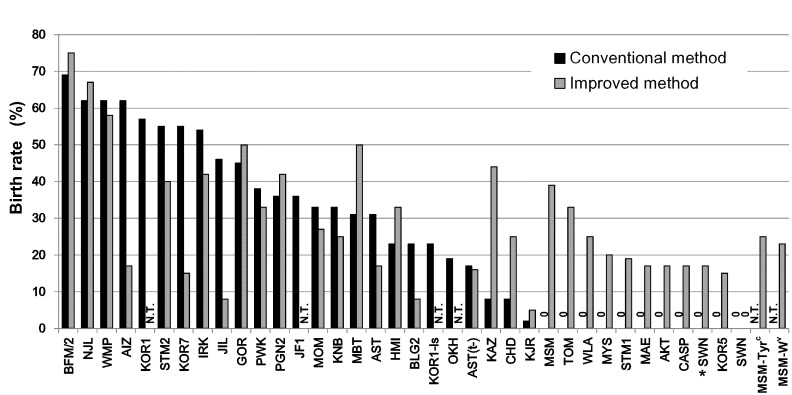
Full-term development of cryopreserved embryos after conventional or improved embryo transfer methods. Living offspring were obtained from all 37 strains with either the conventional (black) or the improved (gray) embryo transfer method. While 10 out of 35 strains failed to produce offspring by the conventional method, all strains tested produced offspring successfully by the improved method (33 strains). Offspring of the SWN strain were born in combination of embryos derived from natural mating and the improved embryo transfer method (asterisk). The detailed data set is available in Table S6. N.T., not tested.

We cryopreserved 2-cell embryos after culture of IVF-derived embryos for 24 h. In most strains (31/37), more than 90% of cryopreserved embryos were morphologically normal after thawing (Figure 4, Table S6), indicating that the high osmolality vitrification (HOV) method was efficient not only for standard laboratory mouse strains [23] but also for wild-derived strains. The vitrified–recovered 2-cell embryos in 35 strains were transferred into oviducts of pseudopregnant ICR females using the conventional method. Normal-looking offspring were obtained at term from 25 (71%) of the strains tested (Figure 5, Table S6). By contrast, the improved embryo transfer method (co-transfer of ICR embryos and CsA injection) was successful in 32/33 (97%) of the strains tested. It should be noted that practically high (15–39%) birthrates could be achieved even in strains in which the conventional embryo transfer method was unsuccessful. Although no offspring from the SWN strain were obtained from IVF-derived embryos by either embryo transfer method, the cryopreserved embryos derived from natural mating developed into offspring (17%, 4/24) using the improved transfer method (Figure 5, Table S6). In the MSM strain, the birthrates per embryo transferred was increased from 29% in our previous study [13] to 39% here. This improvement could be attributable to an increase in the number of transferred embryos from four to nine (excluding three or four ICR embryos) for each oviduct. We also transferred larger numbers of embryos (eight to 15) in the KJR, SWM, AST(t–), and MSM background strains, which are known to be poor responders to embryo transfer stimuli based on our experience and that of other laboratories (unpublished).

### Efficiency of Reproductive Performance

**Figure 6:**
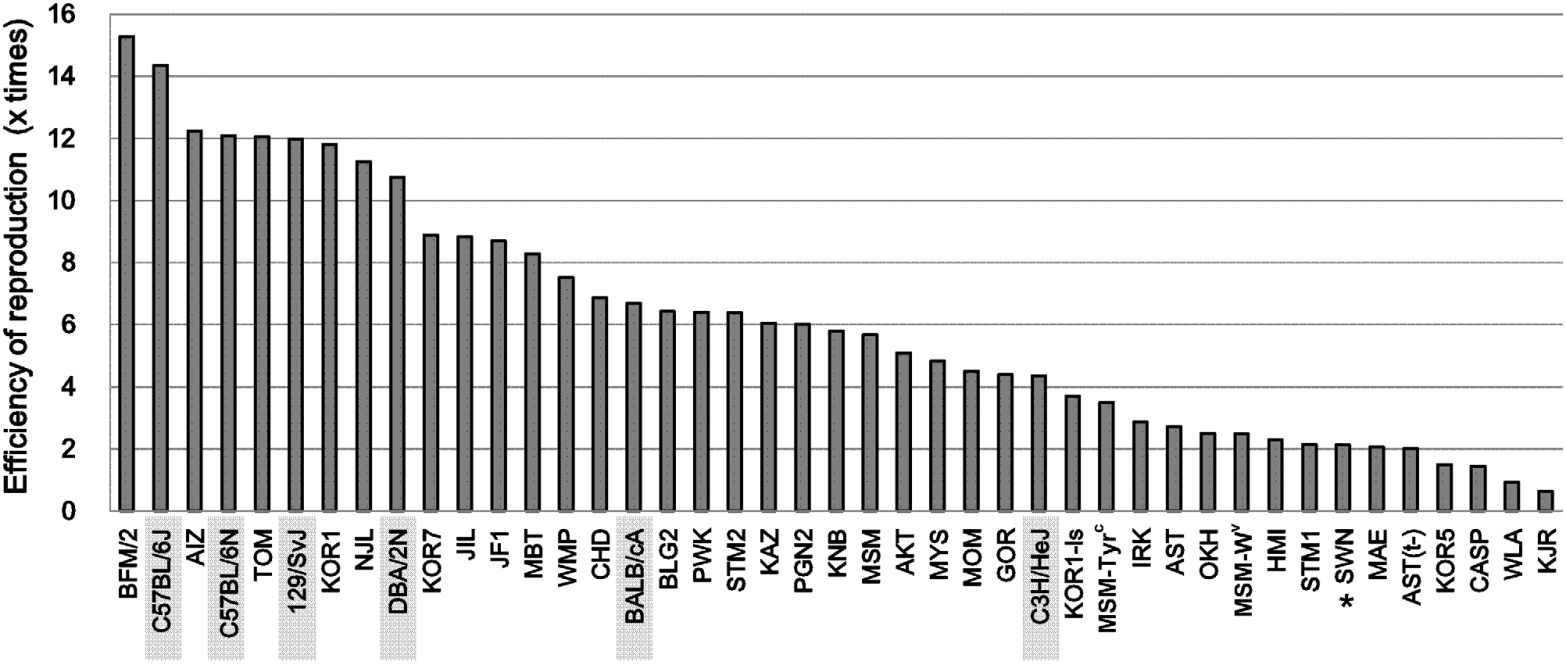
The overall reproduction efficiencies in wild-derived strains and standard inbred strains (shaded). The efficiencies were calculated as the number of offspring produced from one superovulated female; i.e., the multiplication of the averages of the number of collected oocytes per female by eCG or AIS treatment, the fertilization rates with fresh spermatozoa, the survival rates of embryos cryopreserved with HOV method, and the birthrates after embryo transfer by conventional or improved method. For the SWN strain, the birthrate data were obtained from embryos derived from natural mating (asterisk) (see Figure 5). For standard inbred strains, refer to Tables S7 and S8.

Finally, we calculated the overall efficiency of reproduction by ART, namely the expected number of offspring produced from one female based on the mean number of oocytes, fertilization rates, survival rates after cryopreservation, and birthrates after embryo transfer in each strain. In the MSM strain, for example, an overall efficiency of 5.7 offspring per female = 15.6 embryos obtained per female×93% (survival rate after cryopreservation)×39% (birthrate after embryo transfer). The overall production efficiencies for wild-derived strains, together with those of laboratory mouse strains are shown in Figure 6. The data from laboratory strains were updated from those published previously [23] (Tables S7 and S8). From our experience, the mean litter size by natural mating in wild-derived strains is roughly four to six offspring/female. Thus, 22 out of 37 strains (59%) were considered to show efficiencies higher than or comparable (≥4 offspring per female) to those of natural mating. The efficiencies of the remaining 15 strains were poorer (<4), but normal pups were born by our ART techniques in these strains. Although there were considerable strain-specific differences in the overall production efficiencies, a series of ART techniques have been devised for all 37 strains belonging to *Mus musculus*.

## Discussion

This study was undertaken to devise the basic ART for 37 wild-derived strains of mice maintained at the RIKEN BRC. Mouse resource centers including ours have greatly contributed to advancements in biomedicine by collecting, preserving, and distributing mouse resources of high quality [12], [24], [25]. Consisting of several technological steps of gamete/embryo handling, ART now plays a central role in the routine operations of all mouse resource centers. However, every step of ART has been developed mainly for laboratory strains of mice and is not always applicable for wild-derived strains [26]–[28]. In this study, we successfully obtained normal offspring from all strains tested after superovulation, IVF, embryo cryopreservation, and embryo transfer. Although the efficiencies varied greatly with the strain, we consider our results to be a significant step forward for the practical use and preservation of wild-derived strains, which have long been important issues for mouse repositories. We have provided a large set of basic data on superovulation, IVF using fresh or frozen-thawed spermatozoa, embryo cryopreservation, and embryo transfer in this study. The major strain-specific differences appeared in the efficiencies of superovulation and embryo transfer. The IVF protocol using fresh spermatozoa needed slight modifications according to strain. By contrast, our embryo cryopreservation protocol by the high osmolality vitrification (HOV) method was proven applicable to all the wild-derived strains. These comprehensive data on every ART step will help researchers who are going to use wild-derived strains in their research. The data set will also be invaluable for mouse resource centers planning to preserve strains as cryopreserved stocks. Indeed, in our center, we have collected enough numbers of cryopreserved embryos (>300–500, depending on the strain) for termination of reproduction of live mice in 32 out of 37 strains reported in this study.

As described above, great strain-specific differences were observed in responsiveness to the superovulation regimen. Previously, we have reported that AIS instead of eCG treatment significantly increased the number of oocytes by four- and six-fold in MSM and JF1 strains, respectively, which belong to the *M. m. molossinus* group [13]. Here, we found that this characteristic was shared with many other *M. m. molossinus* strains. The *M. m. molossinus* strains are considered to be derived from a cross of *M. m. musculus* and *M. m. castaneus* and are distributed predominantly in East Asia. By contrast, the *M. m. domesticus* strains, which are broadly distributed in Europe, responded better to eCG than to AIS. The major laboratory strains are phylogenetically close to *M. m. domesticus*, but they are not always poor responders to AIS as far as we could examine (unpublished data). As we used wild-derived strains mainly at 5–8 weeks of age, it is possible that female mice out of this age range would respond differently to AIS.

We confirmed that the addition of GSH to the IVF medium, which was developed originally by Bath in 2010 [20], was highly effective in wild-derived strains of mice, especially when frozen-thawed spermatozoa were used. It is likely that this technique would be most useful in producing a large number of embryos at one time for some experiments, such as generating gene-modified mice. A large number of mice from wild-derived strains can also be generated by this IVF protocol for experiments using live animals.

In general, the most efficient method for preserving inbred strains mice is cryopreservation of their embryos in LN_2_. So far, many protocols for cryopreservation of mouse embryos have been developed, but only a few can be applied to a broad range of strains. It is known that slow-freezing protocols have advantages over rapid protocols, with their relatively broader applicability, but they need a special device (a programmed freezer) and take much time. The protocols of embryo vitrification were developed to circumvent these drawbacks associated with such slow-freezing methods. However, the inherent toxicity of the cryoprotective agents and the critical requirement of the super-low temperature condition often damage embryos during equilibration or warming steps especially in some sensitive strains such as BALB/c. We have recently developed an HOV vitrification protocol that can minimize the toxicity of cryoprotective agents and increase the devitrification temperature [23]. In this study, we applied HOV to embryos from many wild-derived strains of mice and confirmed that the survivability (about 80%) of embryos was highly reproducible among the wild-derived strains tested. This suggests unequivocally that HOV might have broad applicability to embryos from many other strains of mice with laboratory- as well as wild-derived genetic backgrounds.

The final step of ART is embryo transfer to recipient pseudopregnant foster mothers and its efficiency might affect the overall production efficiency critically. Before we developed a modified embryo transfer protocol for wild-derived MSM strain in 2012 [13], it was extremely difficult to produce offspring from wild-derived strains after manipulation of gametes/embryos *in vitro* [29]. The modifications comprised immunosuppressive treatment of recipient females with CsA and cotransfer with ICR strain embryos, which enhanced the survival of fetuses. Although it would have been interesting to discriminate the outcomes using different strains of wild-derived strains, because of the limited number of embryos, we employed this combined method to increase the possibility of obtaining live pups. We applied this technique to 33 wild-derived strains of mice belonging to five subspecies. Unlike responsiveness to the superovulation regimen, there seemed to be no subspecies-specific differences in terms of dependency on the embryo transfer protocol. For example, the conventional embryo transfer protocol using untreated ICR females was successfully applicable (>60% birthrates) to the BFM/2, NJL, WMP, and AIZ strains, which belong to *M. m. domesticus*, *M. m. musculus*, *M. m. domesticus*, and *M. m. molossinus*, respectively. This was also the case with the opposite phenotype; 10 strains from which we were unable to produce offspring by the conventional method were derived from five different subspecies. At present, we do not know what factors determine these outcomes, but some genetic factors independent of the subspecies background might regulate interactions between the embryos and the recipient reproductive tract. This might be an interesting issue in the field of reproductive immunology. As we reported before, the MSM and JF1 strains, both of which belong to *M. m. molossinus*, showed distinct characteristics in terms of embryonic development after embryo transfer. They have both common and disparate sequences over their entire genomes when compared with that of laboratory mice such as C57BL/6 [2], [11]. Such genetic information would provide important clues to addressing the question of embryo implantation.

In conclusion, we have successfully devised a series of ART protocols for 37 wild-derived strains of mice belonging to five subspecies groups. Although there is still a need for further refinements in some strains, the large set of data on superovulation, IVF, cryopreservation, and embryo transfer available here will provide much necessary information for biomedical researchers using wild-derived strains, and for mouse resource centers maintaining them as invaluable genetic resources.

## Supporting Information

Table S1 **The mean numbers of oocytes collected per female after eCG or AIS treatment.**
(XLS)

Table S2 **Results of superovulation by eCG or AIS injection in** ***M. m. molossinus*** **.**
(XLS)

Table S3 **Results of superovulation by eCG or AIS injection** ***M. m. musculus*** **.**
(XLS)

Table S4 **Results of superovulation by eCG or AIS injection in** ***M. m. domesticus*** **,** ***M. m. castaneus*** **, and** ***M. m. spp*** **.**
(XLS)

Table S5 **Fertilization rates after IVF with fresh or frozen spermatozoa.**
(XLS)

Table S6 **Survival rates of 2-cell embryos after vitrification by HOV and warming and full-term development of cryopreserved embryos after embryo transfer.**
(XLS)

Table S7 **Results of oocyte collection and in vitro fertilization in standard inbred strains.**
(XLS)

Table S8 **Survivability and developmental ability of vitrified embryos in standard inbred strains.**
(XLS)
